# Development of micro-fibrous solid dispersions of poorly water-soluble drugs in sucrose using temperature-controlled centrifugal spinning

**DOI:** 10.1016/j.ejpb.2016.03.021

**Published:** 2016-06

**Authors:** Stefania Marano, Susan Anne Barker, Bahijja Tolulope Raimi-Abraham, Shahrzad Missaghi, Ali Rajabi-Siahboomi, Duncan Q.M. Craig

**Affiliations:** a UCL School of Pharmacy, 29-39 Brunswick Square, London, UK; b Colorcon Inc., Global Headquarters, 275 Ruth Road, Harleysville, PA 19438, USA

**Keywords:** Centrifugal spinning, Microfiber, Amorphous solid dispersion, Sucrose, Poorly water soluble drug, Dissolution enhancement, Supersaturation

## Abstract

Solid dispersion technology represents a successful approach to addressing the bioavailability issues caused by the low aqueous solubility of many Biopharmaceutics Classification System (BCS) Class II drugs. In this study, the use of high-yield manufacture of fiber-based dispersion is explored as an alternative approach to monolith production methods. A temperature-controlled solvent-free centrifugal spinning process was used to produce sucrose-based microfibers containing the poorly water-soluble drugs olanzapine and piroxicam (both BCS Class II); these were successfully incorporated into the microfibers and the basic characteristics of fiber diameter, glassy behavior, drug loading capacity and drug–sucrose interaction assessment were measured. Scanning electron microscopy revealed that bead-free drug-loaded microfibers with homogenous morphology and diameter in the range of a few micrometers were prepared using our process. Differential scanning calorimetric and X-ray diffraction analyses showed that both drug and carrier were present in the amorphous state in the microfibers, although in the case of piroxicam-loaded microfibers, the presence of small amounts of crystalline drug was observed under polarized light microscopy and in Fourier transform infrared spectra. Drug dissolution performance was evaluated under both sink and non-sink conditions and was found to be significantly enhanced compared to the corresponding crystalline physical mixtures and pure drugs, with evidence of supersaturation behavior noted under non-sink conditions. This study has demonstrated that microfiber-based dispersions may be manufactured by the centrifugal spinning process and may possess characteristics that are favorable for the enhanced dissolution and oral absorption of drugs.

## Graphical abstract

**Figure:**
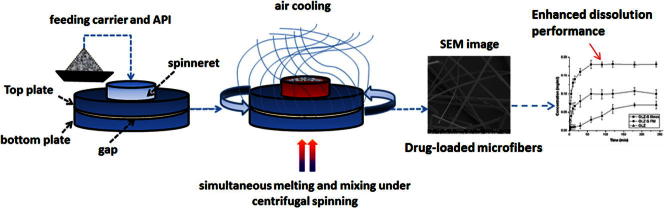

## 1 Introduction

The improvement of drug dissolution performance of poorly water-soluble active compounds remains a key challenge in the field of pharmaceutical technology. High-throughput screening in non-aqueous solutions, the application of combinatorial chemistry as well as the desire to increase drug potency via increasing receptor binding mediated by hydrophobic interactions, have all led to a dramatic increase in new drug candidates with low water solubility [1], [2], [3]. The majority of these compounds belong to class II (low solubility and high permeability/high metabolism) within the Biopharmaceutics Classification System (BCS) and Biopharmaceutics Drug Disposition Classification System (BDDCS). It has been estimated that up to 75% of drugs currently under development will have poor solubility in water, which clearly affects the potential for drug absorption after oral administration [4].

Several approaches have been developed for the enhancement of the dissolution rate and in turn oral bioavailability of poorly water-soluble drugs, including solid dispersions [5], [6], [7]. These are dispersions of one or more ingredients in a solid form of continuous matrix/carrier prepared by solvent, melting or solvent-melting methods. The increase in the drug dissolution rate from solid dispersions has been attributed to several factors, such as reduction in drug particle size, solubilization properties of the carrier material, enhanced wettability and dispersability of the drug by the carrier and conversion to the amorphous form of the drug [8]. In particular, solid dispersions containing the drug in the amorphous state have generally shown to lead to a significant enhancement of dissolution rate compared to formulations containing the drug in the crystalline state, due to the reduction/elimination of the long-range three-dimensional order in the crystal [9]. Despite the great potential of this approach, solid dispersions are still an ‘under-realized’ tool, mostly due to their physical instability and difficulties associated with high cost production as well as up-scaling of the final product [9].

The majority of studies that use solid dispersion technologies have involved the production of monolith systems using methods such as hot melt extrusion (HME); such approaches have potential in terms of both efficiency and feasible scale up to production levels and relevant examples include Isoptin® SRE-240 and Kaletra® tablets (Abbott Laboratories) [10]. Recently, the idea of combining solid dispersion technology and nanotechnology has started to attract interest within the field, notably in terms of the production of solid dispersions in the form of nanofibers using techniques such as electrospinning [11], [12], [13]. Nanofibers have been extensively studied for a wide range of applications, including filtration [14], protective clothing [15], tissue engineering [16] and sensors [17]. Their use in the drug delivery field has increased dramatically over recent years due to evidence for greater drug dissolution enhancement compared to conventional solid dispersion-based formulations, such as the case of electro-spun spironolactone [13] and itraconazole [18]. Indeed, studies to date using electrospinning have suggested that the particular porous structure and high surface area/volume ratio of nanofibers may represent key advantages to overcoming the drug solubility and bioavailability concerns of many drugs. However, the application of electrospinning within the pharmaceutical field has been limited by poor cost–yield efficiency due to the complexity of the production process and low production rates, despite the development of scale-up approaches including multi-nozzle [19], nozzle-free [20] and high speed electrospinning [18].

In this study, we explore the use of an alternative fiber production approach as a means of producing solid dispersions, namely centrifugal spinning. This approach, patented by Hooper in 1924, is a well-known extrusion process which has been widely used for the manufacture of micrometer-scale glass fibers for more than half a century [21], as well as being widely used in the confectionary industry. Centrifugal spinning is a one-step top-down technique, whereby material is placed into a preheated or room temperature rotating metal container (spinning head) with side nozzle holes or gap between two plates, which rotates at high speed (generally in the range of 2000–13,000 rpm). The centrifugal force generated at high rotation speed pushes the spinning fluid through the holes of the spinning head. While the spinning fluid is ejected, it undergoes a stretching process due to air friction force followed by fast air solidification by cooling or solvent evaporation and formation of extruded fibers in the range of nano or micro scale [22], [23]. The centrifugal force ($F_{c}$) and air friction force ($F_{f}$) are described by Eqs. (1), (2) [24]:

(1) $$F_{c}=m\omega_{s}^{2}D_{s}/2$$

(2) $$F_{f}=\pi C\rho A\omega_{j}^{2}D_{j}^{2}/2$$

where m is the mass of the molten material, C is a numerical drag coefficient, ρ is the air density, and Ais the cross sectional area of the jet; $\omega_{s}$, $D_{s}$, $\omega_{j}$, and $D_{j}$, are the rotating speed and diameter of the spinneret and the jet respectively. Therefore, centrifugal and air friction forces increase as the rotation speed increases, which in turn leads to greater stretching of the liquid jet and reduction in fiber diameter.

Many studies have shown that centrifugal spinning is a valid alternative approach to producing nanofibers from a wide range of materials at high rate and low cost due to simple equipment set-up, solvent-free capability and no need of use of high voltage [24]. Relevant examples are Forcespinning™ [25], rotary jet spinning [26] and pressurized gyration [27]. It is also noteworthy that during the centrifugal spinning process, materials are spun very quickly (a few seconds); therefore, the brief residence time of material exposed to high temperatures should reduce the risk of material degradation [28]. It is therefore logical to explore the use of centrifugal spinning processes as a means of producing solid dispersions, given the high surface area generated which may potentially further enhance dissolution advantages. Table 1 compares the technological aspects of the most common methods for the manufacture of solid dispersions with electrospinning and the centrifugal spinning process [25], [26], [27], [29], [18], [30], [31], [32], [33], [34].

**Table 1:** Summarized technological aspects of the most common methods of solid dispersion preparation compared to the electrospinning and centrifugal spinning processes.

Method	Production rate (lab-scale)	Material choice	Method used	Disadvantage	Application	Ref.
Centrifugal spinning	300 g to 6 kg/h	Broad	Melting/solvent evaporation	Broad range of fiber diameter	Fiber glass production, oral thin films, tissue engineering scaffolds	[25], [26], [27], [29]

Electro-spinning	0.2–450 g/h	Broad but dependent on intrinsic properties of polymer fluid	Mainly solvent evaporation, melting	Low production rate, applied high voltage, jet stability	Filtration, tissue engineering, protective clothing, energy storage, manufacture of solid dispersions	[18], [30]

Hot melt extrusion	100 g to 2 kg/h	Broad but dependent on thermoplastic properties of polymers	Melting	Not suitable for thermally labile drugs	Rubber/plastic fabrication, pelletized feeds, implants, injection molding manufacture of solid dispersions	[31], [32]

Spray drying	Lower that hot melt extrusion	Soluble in the solvent of choice	Solvent evaporation	Potential solvent residue	Pharmaceutical materials processing, food industry, paint pigments, ceramic materials, catalyst supports, manufacture of solid dispersions	[33]

Freeze drying	Lower than hot melt extrusion and spray drying	Soluble in the solvent of choice	Solvent evaporation	Potential solvent residue, time consuming	Food industry, treatment of heat sensitive materials manufacture of solid dispersions	[34]

Inspection of this table indicates that nanofibers can be produced at high rates using the centrifugal spinning method for various applications. This leads to the suggestion that the use of this approach could be extended into the pharmaceutical industry for the preparation of solid dispersions. However, perhaps surprisingly, studies to date on drugs incorporated into nanofibers by centrifugal spinning are still very limited. One notable approach has been the production of Flash Dose® tablets using Shearform™ technology patented by Fluiz in 1989, whereby partially recrystallized sugar (used as a carrier) is milled and blended with unprocessed hydrophilic active compounds and subsequently compressed into fast disintegrating tablets [29]. However, that process differs from the one outlined here, whereby we investigate the production of fully amorphous solid dispersions containing hydrophobic drugs for dissolution enhancement.

In order to explore the feasibility of the centrifugal spinning process for producing solid dispersions in the form of fibers, we describe an inexpensive laboratory scale centrifugal spinning device involving temperature controlling and calibrating a commercial ‘cotton candy’ machine. In this proof-of-concept study, we aimed to demonstrate that the use of the centrifugal spinning approach is an efficient and reliable alternative approach to produce solid dispersions with enhanced dissolution performance of two BCS Class II model drugs, olanzapine (OLZ) and piroxicam (PRX), using sucrose as a carrier. Olanzapine is approved for treatment of psychoses, while piroxicam is a nonsteroidal anti-inflammatory drugs (NSAIDs) used to treat pain associated with arthrosis. Both drugs are administered orally as a single dose of 20 mg/day and have shown very low oral bioavailability due to their poor solubility in water: these are around 43 mg/L for OLZ (dibasic drug with p*K*a values of 5.0 and 7.4) [35] and 14 mg/L for PRX (ampholytic drug with p*K*a values of 5.1 and 1.9) [36]. Besides the low solubility in water, these drugs were chosen on the basis of their thermal stability at the processing temperatures used in this study as supported by other authors [37], [38]. The performance of the final products was evaluated, not only in terms of their ability to enhance the drug dissolution rate (tested under sink conditions), but also in terms of their ability to generate and maintain the supersaturated state (tested under non-sink conditions), as the latter has shown to be crucial for effective absorption of poorly water soluble drugs [39], [40], [41]).

## 2 Materials and methods

### 2.1 Materials

Crystalline olanzapine (*M*_w_ = 321.43 g mol^−1^) was purchased from Myjoy Ltd. (India); crystalline piroxicam (*M*_w_ = 331.34 g mol^−1^) was purchased from Afine Chemicals Limited (China); crystalline sucrose was obtained from Sigma–Aldrich Co. (USA). All buffer salts used for the dissolution medium, as well as dimethyl sulfoxide (DMSO), used for drug loading efficiency measurements, were obtained from Sigma–Aldrich (Germany). All other chemical reagents were of analytical grade.

### 2.2 Methods

#### 2.2.1 Device optimization and calibration

**Fig. 1:**
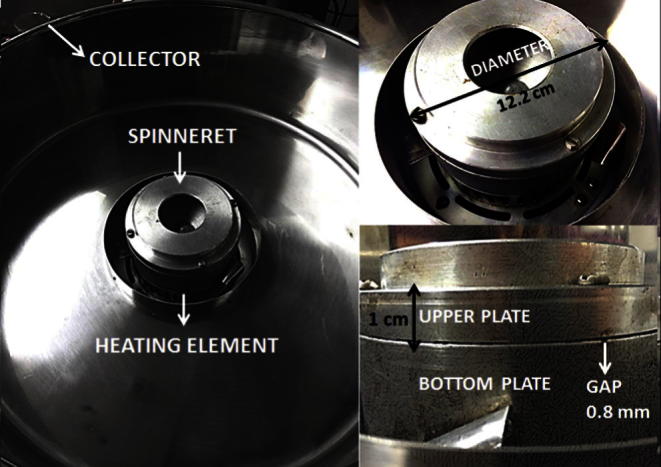
Schematic image of the centrifugal spinning device including dimensional measurements.

As illustrated in Fig. 1 the purchased commercial centrifugal spinning device (ET-MF01 professional, Monster Group, UK) includes three main parts: an aluminum spinneret (12.2 cm diameter) that rotates about its axis, a heating element and a collector. The spinneret consists of two main parts: a bottom plate having a concave cavity configured to receive materials and an upper plate (2 mm gauge) configured to cover and enclose the concave cavity such that a gap of 0.8 mm exists between the two plates. Since the gap is crucial for the consistency of fiber quality and size, the upper plate was replaced with a thicker 1 cm gauge aluminum plate to ensure better control of the gap and facilitate the upper plate removal and cleaning. Once the material is melted and the rotating speed reaches a critical value, simultaneous centrifugal and air friction forces overcome the surface tension of the molten material which is ejected through the gap and stretched into fibers with decreased surface area.

The centrifugal spinning device was provided with both fixed rotating speed (2400 rpm, measured using Smiths Industrial Instruments Ltd. tachometer, UK) and heating temperature (∼200 °C), as this equipment is generally set to spin sucrose only. In order to vary the temperature, the machine was modified by adding an external voltage regulator in the range of 0–250 V. Subsequently, temperature–voltage calibration was performed to establish the exact temperature range in which the machine can operate. This was carried out by gradually increasing the voltage from 0 to 250 V and measuring the temperature at every increment using a temperature sensor (Testo Mini surface thermometer, ±1 °C accuracy and 0.1 °C resolution). Measurements were conducted at least in triplicate.

#### 2.2.2 Sample preparation

**Fig. 2:**
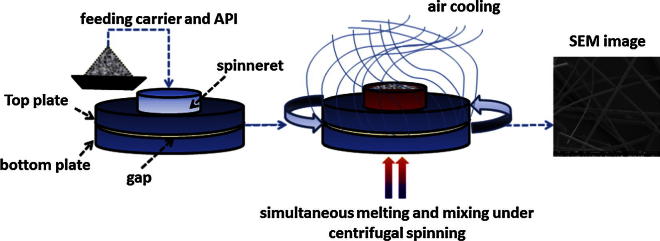
Schematic representation of the centrifugal spinning apparatus and individual process steps in the preparation of drug-loaded microfibers.

Unloaded and 10% (w/w) drug-loaded sucrose microfibers were prepared as described in Fig. 2 using our modified device. Physical mixtures (PM) were prepared by mixing sucrose (90 w%) and drug (10 w%) in a mortar for 5 min. 10 g of starting material were accurately weighed and placed into the spinneret which was preheated to the required temperature. Spinning operations were conducted with a rotational speed of 2400 rpm at room temperature (25 ± 5.0 °C). Fibers formed were collected and characterized within 24 h of preparation.

#### 2.2.3 Yield and drug loading efficiency

The percent yield of drug-loaded microfibers was determined by using Eq. (3).

(3) $$\text{Yield}\;\left(\%\frac{\text{w}}{\text{w}}\right)=\frac{\text{weight of prepared solid dispersion}}{\text{weight of drug}+\text{carrier}}\times 100$$

Drug Loading Efficiency (DLE) is defined as follows in Eq. (4):

(4) $$\text{DLE}\;\left(\%\frac{\text{w}}{\text{w}}\right)=\frac{\text{amount of drug measured}}{\text{theoretical amount of drug based on drug loading}}\times 100$$

Drug loading efficiency was measured by dissolving 10 mg of fibers in 5 mL of DMSO, in which both drug and carrier are soluble, followed by dilution in phosphate buffer (pH: 6.8) for UV detection. The amount of drug was calculated using their respective calibration curves.

#### 2.2.4 Physico-chemical analysis of microfibers

##### 2.2.4.1 Scanning electron microscopy (SEM)

Fiber morphology and diameter were analyzed using a FEITM Quanta 200F Field Emission SEM. Samples were coated with 20 nm of gold under vacuum using a Quorum Q150T Turbo-Pumped Sputter Coater. Data were collected over a selected area of the surface of samples. The average diameter and the percentage frequency of the microfibers were determined from 100 individual measurements using ImageJ (USA, version 1.46r). Diameter distribution graphs were plotted and analyzed using OriginPro 8.0.0.

##### 2.2.4.2 Thermal characterization

Differential scanning calorimetry (DSC) traces were obtained using a TA Instruments Q2000 (Newcastle, DE, USA) with a refrigerated cooling system attached to a dry nitrogen sample purge flow at 50 mL/min. Temperature calibration was performed using indium, n-octadecane and tin at both 2 and 10 °C/min heating rates; heat capacity constant calibration was performed using aluminum oxide TA sapphire disks at 2 °C/min with ±0.212 °C modulation amplitude and 60 s period. Melting temperature (*T*_m_) values were obtained using conventional DSC in which samples were weighed (4–5 mg) into pinholed aluminum pans (Perkin Elmer) and heated from 0 to 250 °C at 10 °C/min. Glass transition (*T*_g_) values were obtained using modulated temperature DSC (MTDSC) from a heat–cool–heat cycle method at 2 °C/min underlying heating rate with amplitude ±0.212 °C and period 60 s. The *T*_g_ was measured in the reheating cycle and determined as the fictive glass transition temperature using the TA Instruments proprietary software Universal Analysis.

Water content and thermal decomposition temperature (*T*_deg_) of both raw materials and formulations were measured using thermogravimetric analysis (TGA) with a Q5000 (TA Instruments, Newcastle, DE, USA). Samples were heated from room temperature up to 100 °C with a heating rate of 10 °C/min and held isothermally for at least 15 min before continuing the heating ramp up to 300 °C. The amount of water content was quantified as the percentage of mass loss observed in the temperature region below the onset of degradation.

Hot stage microscopy (HSM) experiments were conducted on a Leica DML52 microscope with 10–20× magnification lens connected to a FP5/FP52, Mettler Toledo Instruments heating stage unit and a FP90 Mettler Toledo Instruments central processor unit. HSM was mainly used to add further confidence to the data obtained using DSC and TGA. The crystallinity of the samples was evaluated based on the presence or absence of birefringence under polarized light at room temperature. Thermal events were recorded using a JVC color video camera with Studio86 Design capture software.

All measurements were run in triplicate.

##### 2.2.4.3 X-ray powder diffraction (XRPD)

Ambient X-ray powder diffraction (XRPD) measurements were performed using a MiniFlex diffractometer (RigaKu, Tokyo, Japan). Samples were lightly pressed into 20 mm aluminum sample trays and the surface was scraped evenly using a glass slide. A Cu Kα radiation point source (*λ* = 1.5148 Å) was operated at 40 mV and 15 mA. XRPD patterns were recorded using diffraction angles (2*θ*) from 10° to 50° (step size 0.05°; time per step 0.2 s). Data was exported and analyzed using OriginPro 8.0.0. All experiments were conducted in triplicate.

##### 2.2.4.4 Attenuated total reflectance-Fourier transform infrared (ATR-FTIR)

Characterization of fiber molecular structure was performed using attenuated total reflectance Fourier transform infrared spectroscopy (ATR-FTIR) (Bruker Vertex 90 spectrometer). Measurements were performed with a resolution of 2 cm^−1^, 32 scans over 4000–700 cm^−1^ range at room temperature (25 °C). Spectra were analyzed using Opus software version 7.2 and OriginPro 8.0.0. All experiments were conducted in triplicate.

#### 2.2.5 In-vitro dissolution studies

##### 2.2.5.1 Determination of saturation solubility

The thermodynamic solubility (*C*_s_) is defined as the concentration of a compound in a saturated solution when an excess of non-dissolved thermodynamically stable crystals of the compound is in dynamic equilibrium with its solution. To evaluate the increase in thermodynamic solubility of OLZ and PRX from the prepared solid dispersions, saturation solubility measurements were conducted and compared with those of pure drugs alone and in the presence of increasing concentrations of sucrose (from 0.5 to 5 mg/mL), according to the method described by Higuchi and Connors [42]. Known excess amounts of material were added to glass tubes containing 10 mL of phosphate buffer, pH 6.8. Saturation was confirmed by the presence of undissolved material. Saturated solutions were stirred in a shake incubator (SciQuip Mini Incu Shake, UK) at 100 rpm at 37 ± 0.5 °C for 48 h in order to achieve equilibrium. Samples of 1 mL were withdrawn, filtered through a 0.22 μm Millipore Millex® GT filter and properly diluted for UV measurements. All solubility determinations were performed in triplicate. The association constant (ka) was also calculated using Eq. (5), derived from the slope and intercept of phase solubility diagram, whereby concentration of drug dissolved (mol/L) is plotted against the increasing concentration of sucrose (mol/L).

(5) $$\mathrm{ka}=\frac{\text{slope}}{\text{intercept}(1-\text{slope})}$$

##### 2.2.5.2 In-vitro dissolution study under sink conditions

In-vitro dissolution tests under sink conditions were carried out using a USP type II paddle apparatus in 500 mL of phosphate buffer (pH: 6.8) held at a temperature of 37 ± 0.2 °C with paddle speed of 100 rpm. OLZ and PRX both show pH-dependent solubility. Since changes in pH will affect the degree of ionization of such drugs, and hence their solubility, buffer solutions are generally required to keep the pH constant. Therefore, for the preliminary study, phosphate buffer (pH: 6.8) was chosen as a model dissolution medium. The particular pH resembles healthy saliva in the oral cavity and was chosen on the basis of potential application of these formulations as orally disintegrating tablets. Samples equivalent to 10 mg of drug were encapsulated, without any pre-treatment, in size 4 gelatin capsules (Qualicaps Europe SA, Madrid) with onset dissolution of 3 min. At predetermined time intervals, 10 mL samples were withdrawn from each vessel using a 10 mL syringe and replaced with fresh medium to maintain a constant total dissolution volume. The drug concentration in the dissolution medium was measured using UV detection and the release profile plotted as percentage of cumulative drug release versus time using OriginPro 8.0.0. No interference from sucrose or gelatin capsules on the drug assay was observed at the detection wavelengths. For comparison, dissolution behavior of both pure drugs and corresponding PMs at respective formulation ratios were also studied. The particle sizes of both PMs and pure drugs were kept constant by sieving and selecting a 63–125 μm particle size range. All experiments were performed at least in triplicate and the average values and standard deviations were calculated to plot the dissolution profiles.

##### 2.2.5.3 In-vitro dissolution study under non-sink conditions

Dissolution–supersaturation profiles of the formulations were obtained by non-sink dissolution tests in phosphate buffer (pH: 6.8) using a shaking incubator. Samples containing 10 mg of drug were loaded into 50 mL of dissolution medium. 1 mL samples were withdrawn at pre-determined time intervals and filtered through a 0.22 μm Millipore Millex® GT filter. The withdrawn volume was replaced with the same amount of blank dissolution medium from a separate vessel that had also been held at a temperature of 37 ± 0.2 °C. The absorbance of the filtrate was measured by UV after appropriate dilution. Dissolution tests were performed for 4 h, which is approximately the intestinal transit time in humans [43].

## 3 Results and discussions

### 3.1 Preparation of centrifugal-spun microfibers

In order to vary the temperature and to allow the spinning of sucrose in combination with OLZ and PRX, the centrifugal spinning equipment was modified by adding an external voltage regulator and temperature calibration performed. As for all extrusion techniques using heat, the optimal process temperature should be carefully determined on the base of melting (*T*_m_) and degradation (*T*_deg_) temperature values of raw materials alone and in their physical mixtures (PMs) to avoid material degradation or incomplete mixing of the drug in the carrier [28], [44]. Table 2 shows the thermal properties of OLZ, PRX and their PMs with sucrose obtained from DSC and TGA results.

**Table 2:** Experimental thermal properties of raw materials measured by DSC and TGA and corresponding process temperatures used for the preparation of microfibers.

Material	Melting temperature (°C) ± SD	Degradation temperature (°C) ± SD	Water content (%)	Process temperature (°C)
OLZ	196 ± 0.4	269.7 ± 3.0	0.4 ± 0.12	–
PRX	203 ± 0.2	251.7 ± 2.0	0.04 ± 0.01	–
Sucrose	191 ± 0.5	230.2 ± 4.1	0.72 ± 0.2	197
OLZ–sucrose PM	S: 190 ± 0.2 OLZ: 193.1 ± 0.3	234.05 ± 1.0	0.14 ± 0.08	200
PRX–sucrose PM	S: 187.1 ± 0.4 PRX: 199 ± 0.6	232.02 ± 3.2	0.1 ± 0.07	205

HSM images were also collected during heating to add further confidence to the DSC data and to visualize complete melting of each sample. As an example, Fig. S1 shows images of PRX–sucrose PM captured during heating at 10 °C/min from 30 °C up to the temperature when complete melting was observed. According to the HSM images, complete melting of sucrose, OLZ and PRX in the PMs was observed at 197, 200, and 205 °C respectively, which were slightly higher than *T*_m_ values observed in the DSC traces. In addition, the HSM melting temperature values were found to be distinct from the degradation temperature values measured using TGA, observed at 230, 234 and 232 °C for sucrose, OLZ–sucrose and PRX–sucrose PMs respectively and therefore chosen as optimal spinning temperatures. Using these process conditions, unloaded and 10% (w/w) drug-loaded sucrose microfibers were successfully spun using our modified centrifugal spinning device, with relative high yield (85 ± 5%) and drug loading efficiency (91 ± 5.2% for OLZ and 79 ± 4.3% for PRX).

### 3.2 Study of surface morphology

**Fig. 3:**
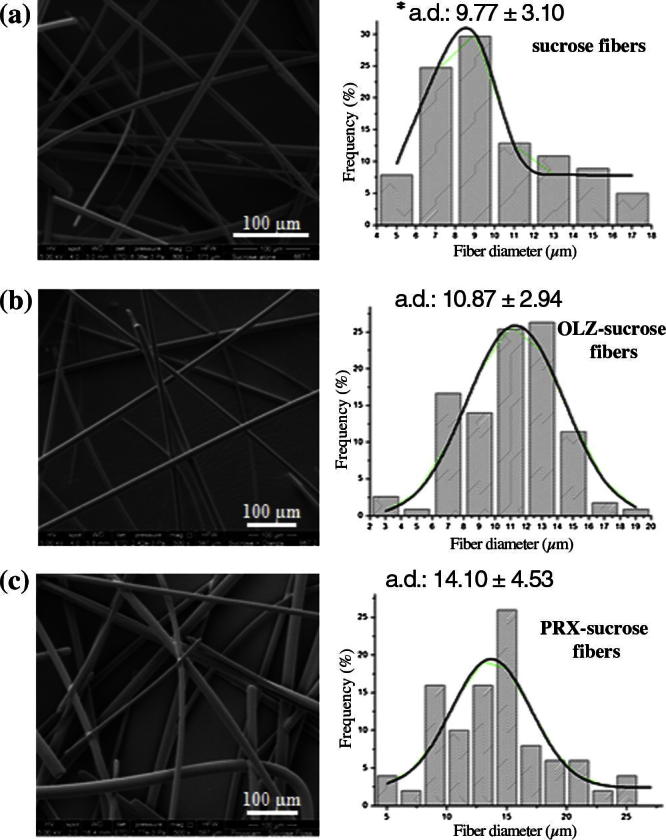
SEM images of the surface morphology (500× magnification) and microfiber diameter frequency diagram of (a) unloaded sucrose microfibers, (b) 10% (w/w) OLZ-loaded sucrose microfibers and (c) 10% (w/w) PRX-loaded sucrose microfibers. ^∗^a.d. = average diameter calculated using about 100 individual diameters for each sample.

Fig. 3 shows the surface morphology of pure sucrose microfibers, and 10% (w/w) OLZ- and PRX-loaded sucrose microfibers, alongside the corresponding microfiber diameter frequency diagrams for each formulation. All formulations show consistent morphology, a relatively small variation in diameter, smooth surface and random orientation. Moreover, in the case of OLZ- and PRX-loaded microfibers, the homogeneous morphology and the lack of visible drug crystals initially suggest that both drugs are molecularly distributed within sucrose microfibers. No statistical difference in microfiber diameters was found between sucrose (mean value 9.77 μm) and OLZ-loaded sucrose microfibers (mean value 10.87 μm), whereas a slightly greater diameter was found for PRX-loaded sucrose microfibers (mean value 14.10 μm). This might be attributed to the lack of drug–carrier interactions and the presence of drug molecules entrapped in the free volume available in the amorphous sucrose matrix [45].

### 3.3 Characterization of physical structure

**Fig. 4:**
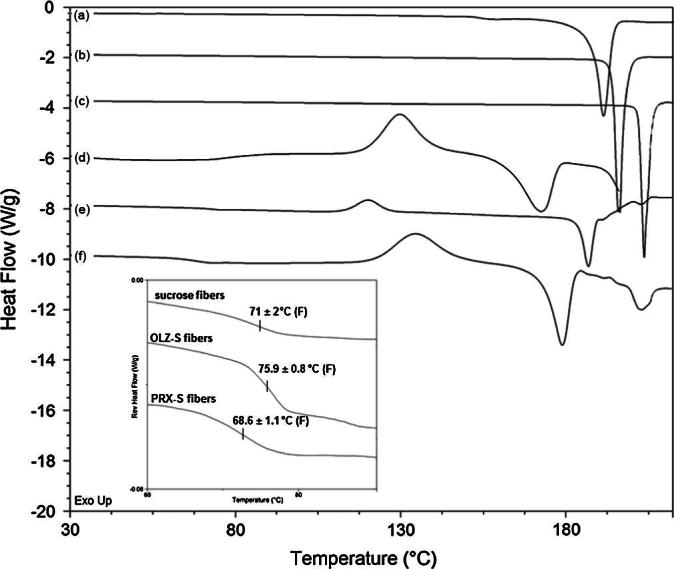
MTDSC total heat flow traces of raw materials: (a) sucrose, (b) OLZ and (c) PRX; and microfibers: (d) sucrose, (e) 10% (w/w) OLZ-loaded sucrose microfibers and (f) 10% (w/w) PRX-loaded sucrose microfibers with inset view showing magnification of glass transitions analyzed with reversing heat flow.

The diffractograms collected from crystalline raw materials and microfibers are displayed in Fig. S2. As expected, OLZ, PRX and sucrose raw materials have a crystalline nature, with sharp intense peaks throughout their diffraction patterns. However, all freshly prepared microfibers showed relatively broad halo patterns suggesting only short-range order due to the conversion from the crystalline to the amorphous state. The amorphous state was also confirmed by studying the thermal behavior of the formulations using MTDSC. The MTDSC traces of raw materials and drug-loaded sucrose microfibers are shown in Fig. 4. Sharp endothermic melting peaks for pure sucrose, OLZ and PRX observed at ∼191, 196 and 203 °C respectively clearly indicate their crystalline nature. After the spinning process, all microfiber formulations were confirmed to be in the amorphous state by the presence of a single *T*_g_, followed by exothermic crystallization and endothermic melting peaks. *T*_g_ values were observed at ∼71, 76, and 68 °C for sucrose, OLZ–sucrose and PRX–sucrose microfibers respectively. Moreover, the presence of a single *T*_g_ for both drug-loaded formulations suggests miscibility of the two components at the drug–carrier ratio under investigation. Interestingly, after exothermic recrystallization of drug-loaded microfibers, no melting peak of OLZ was observed while such a peak was observed for PRX at 202.3 °C (judged to be Form I from comparison with literature data). However, equivalent solid dispersions, prepared using DSC by melting respective PMs to just above their melting point and quenching quickly, did not show any melting endotherm of PRX indicating that both drugs were molecularly incorporated in the amorphous state within the sucrose matrix (data not shown). Therefore, this observation suggests that either the very fast centrifugal spinning process did not allow complete melting of PRX during microfiber manufacture or else the PRX partially recrystallized from sucrose microfibers between manufacture and analysis. It is worth mentioning that drugs themselves as well as the presence of water may act as plasticizers, thereby increasing the possibility of drug or carrier recrystallization compared to the carrier alone [46], which may be the case here with PRX. In contrast, the absence of an OLZ melting peak in the microfibers upon heating suggests that OLZ was molecularly dispersed within the sucrose microfibers and remained so during the manufacturing and testing processes.

**Fig. 5:**
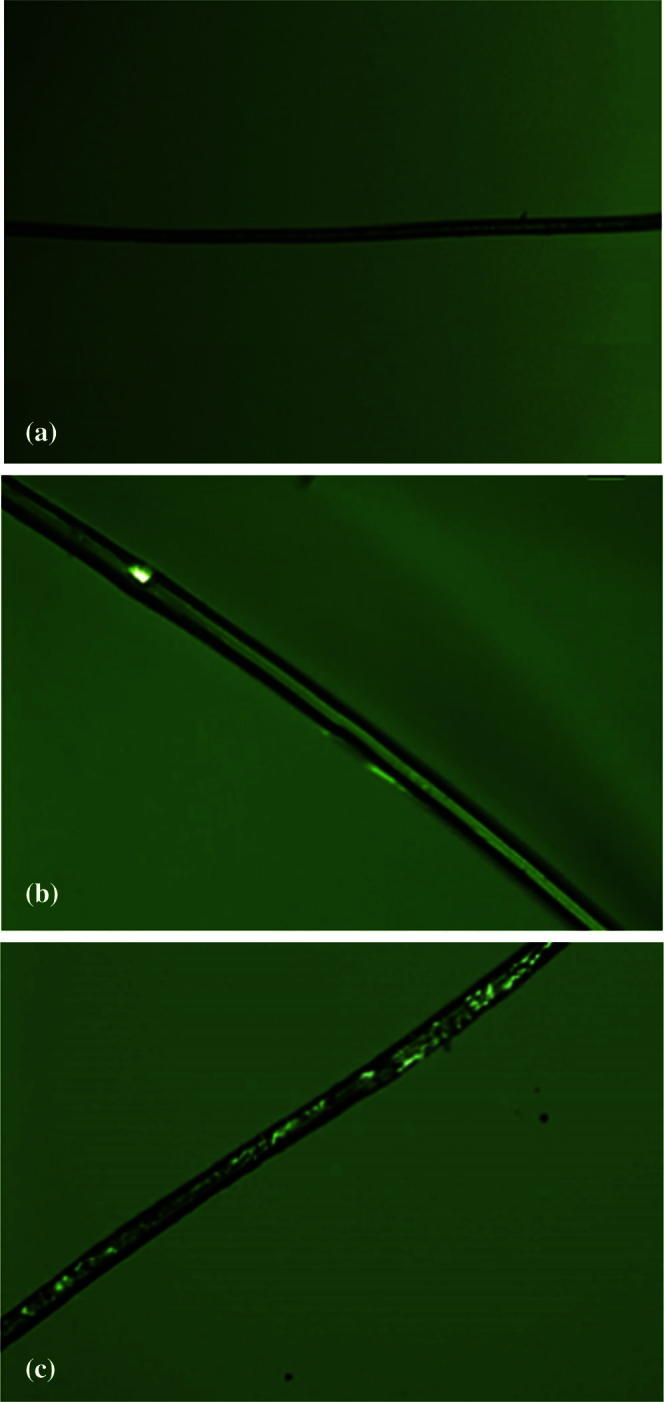
Microscopic images at 20× magnification of freshly prepared microfibers: (A) unloaded sucrose, (B) 10% (w/w) OLZ-loaded sucrose, (C) 10% (w/w) PRX-loaded sucrose.

In order to obtain additional information about the physical state of freshly prepared microfiber formulations, polarized light microscopic images of samples were taken at room temperature. Crystalline and amorphous forms can be distinguished based on the presence or absence of sample birefringence when observed under polarized light. Birefringence is generally an indication of crystallinity whereas amorphous forms show no birefringence. Images of freshly prepared unloaded and drug-loaded microfibers are shown in Fig. 5. No birefringence was observed for unloaded sucrose microfibers, indicating an amorphous form, which was in agreement with XRPD and DSC results. In contrast, birefringence was seen in PRX-loaded microfibers and only a trace in OLZ-loaded microfibers, suggesting the presence of some crystalline material which was not detected using XRPD. Overall therefore both systems are very largely amorphous, although there is evidence for the trace presence of crystals, particularly for PRX.

### 3.4 Spectroscopic characterization of fiber structure

ATR-FTIR is used in this study to further elucidate the physical state and molecular structure of microfibers as well as identify potential drug–carrier molecular interactions occurring as a result of simple mixing or processing at relatively high temperature. FTIR spectra of pure sucrose, OLZ, PRX and drug loaded microfibers for OLZ and PRX are shown in Fig. S3. In addition, Table 3 summarizes ATR-FTIR absorption peak assignments for the drugs alone and in their PMs with sucrose and 10% w/w drug-loaded microfibers. The molecular structures of most stable polymorphs for OLZ (Form I) [47] and PRX (Form I) [48] have already been fully elucidated in previous studies using ATR-FTIR. The PRX raw material spectrum shows characteristic peaks (Form I) at 3338, 1628, 1575, 1525 and 1179 cm^−1^ assigned to the ν(O–H) vibrations band, ν(C=O) of the amide, ν(C=N) of the pyridyl nitrogen and ν(N–H) of the tertiary amine and symmetric vibration of SO_2_, respectively. OLZ raw material exhibits characteristic peaks (Form I) at 1582 cm^−1^ ν(C=N) assigned to the azepine ring, 1556 cm^−1^ ν(C=C) of the benzene and thiophene rings and 1411 cm^−1^ due to deformation of methyl. Peaks in the region between 1600 and 1500 cm^−1^ were chosen to identify the presence of the drugs in the formulations as sucrose shows virtually no absorption in this region. The O–H stretching mode bands of sucrose raw material are observed at 3566, 3391, and 3339 cm^−1^ and corresponding O–H deformation mode bands at 1238, 1209, 1161, and 1342 cm^−1^ [49].

**Table 3:** ATR-FTIR absorption peak assignments for model drugs alone and in their PMs with sucrose and 10% w/w drug loaded microfibers.

Absorption band (cm^−1^)			Assignment [47], [48], [49]
OLZ	OLZ–sucrose PM	OLZ–sucrose microfibers	
2935	2936	2929	C–H stretching
2838	2838	–	CH_2_ symmetric stretching
1582	1583	1588	C=N stretching
1556	1556	1560	Contribution from C=O and C–N stretching
1142	1143	–	Aromatic ring stretching
1411	1409	1406	CH_3_ deformation
1224	1224	1218	C–N stretching

PRX	PRX–sucrose PM	PRX–sucrose microfibers	
3338	3339	–	–OH, –NH stretching
1628	1629	1630	C=O amide carbonyl group
1575	1575	1575	C=N stretching pyridyl nitrogen
1525	1527	1524	C=C stretching of the benzene and thiophene rings
1179	1180	1180	Symmetric vibration of SO_2_

ATR-FTIR spectra of PMs of both drugs with sucrose revealed a summation of the individual ATR-FTIR spectra of the respective components with no apparent wavenumber shift of the peaks (data not shown). This suggests a lack of interaction between drugs and sucrose in their PM systems. Characteristic peaks of OLZ and PRX were found in microfiber formulations, confirming the presence of the drugs in the samples under investigation. The O–H stretching mode bands of sucrose raw material disappeared in the pure sucrose microfiber spectrum, suggesting a lack of order in the sucrose molecular structure due to amorphization, as previously shown from DSC and XRPD results.

**Fig. 6:**
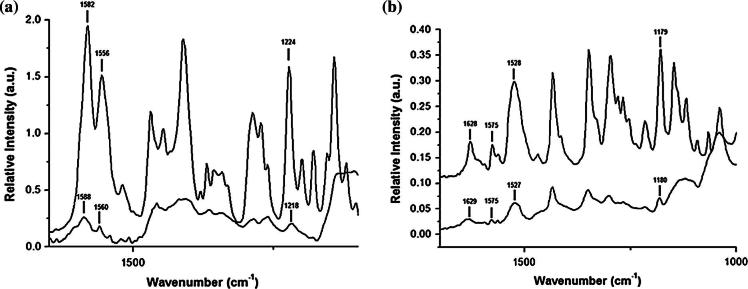
ATR-FTIR spectra in the region between 1700 and 1000 cm^−1^ of (a) crystalline OLZ (top), OLZ–sucrose microfibers (bottom) and (b) crystalline PRX (top) and PRX–sucrose microfibers (bottom).

Interactions between a drug and a carrier such as hydrogen bonding or hydrophobic interactions are often considered to be crucial for producing high quality solid dispersions with acceptable stability [50]. Sucrose molecules contain free hydroxyl groups which could act as potential proton donors for hydrogen bonding with proton acceptor sites present in both OLZ (mainly tertiary amines from azepine and piperazinyl rings) and PRX (mainly SO_2_, amide groups and tertiary amines) molecules. Fig. 6a and b shows ATR-FTIR spectra of OLZ–sucrose and PRX–sucrose microfibers at the finger print region of interest. In both formulations, characteristic peaks of the drugs were found to be fewer in number and broader than in the spectra of the raw materials, due to the conversion from the crystalline to the amorphous state. In the case of OLZ–sucrose microfibers, the shift to higher wavenumbers of the peaks at 1582 and 1556 cm^−1^ assigned to ν(C=N) (azepine ring) and ν(C=C) (benzene and thiophene rings) respectively might be associated with the weakening of OLZ structure due to amorphization, whereas the shift of ν(C–N) to lower wavenumber from 1224 to 1118 cm^−1^ may suggest a hydrogen bonding interaction between OLZ and sucrose. In contrast, no chemical shifts were observed in the case of PRX–sucrose microfibers although characteristic peaks were dramatically lowered in intensity. This might indicate that low level of crystalline PRX was preserved after processing. This would be in agreement with the presence of the melting peak of PRX in the DSC scans and the observation under microscopy of microcrystalline PRX particles within the sucrose matrix.

### 3.5 Dissolution study

#### 3.5.1 Phase solubility

Highly hydrophilic excipients such as sugars are well-known carriers used for solid dispersion-based formulations [51], [52], [53], [54]. In order to examine the aqueous solubility of the model drugs as well as the potential solubilizing power of sucrose, the equilibrium solubility of OLZ and PRX was measured in the presence and absence of sucrose at increasing concentrations (from 0.1 to 5 mg/mL) according to Higuchi and Connors’s method [42]. The association constants (ka) and other relevant parameters used in the phase solubility study are shown in Table 4. Pure OLZ and PRX show similar solubility values in the absence of sucrose (0.068 and 0.073 mg/mL, respectively). The solubility of both drugs increased as a function of sucrose concentration almost linearly (*R*^2^ = 0.95–0.97) over the entire concentration range (0.1–5 mg/mL) (see Fig. S4). The linearity of the phase solubility diagram with slopes lower than one (∼0.015–0.02) for both drugs and relatively high association constants (68.33 and 101.52 M^−1^ for OLZ and PRZ, respectively) may indicate potential formation of 1:1 stoichiometric water soluble complexes due to hydrogen bonding interactions between both drugs and sucrose [55], [56]. It is therefore evident that sucrose has some influence on the solubility of the drugs which may contribute to any subsequent dissolution behavior.

**Table 4:** Equilibrium solubility of pure OLZ and PRX in phosphate buffer (PBS) (pH: 6.8) at 37 °C in the presence and absence of increasing concentrations of sucrose (from 0.1 to 5 mg/mL) and corresponding association constants (*ka*).

Added sucrose (mg/mL)	Solubility of OLZ ± SD in PBS (pH: 6.8) (mg/mL)	Solubility of PRX ± SD in PBS (pH: 6.8) (mg/mL)
Without sucrose	0.069 ± 0.005	0.073 ± 0.0052
0.1	0.073 ± 0.0049	0.079 ± 0.0045
0.5	0.080 ± 0.0031	0.083 ± 0.0025
2	0.101 ± 0.0029	0.112 ± 0.0047
3	0.127 ± 0.0062	0.155 ± 0.0056
5	0.14 ± 0.0042	0.173 ± 0.006
ka at 37 °C (M^−1^)	68.326 (*R*^2^ = 0.971)	101.52 (*R*^2^ = 0.955)

#### 3.5.2 Dissolution study under sink conditions

**Fig. 7:**
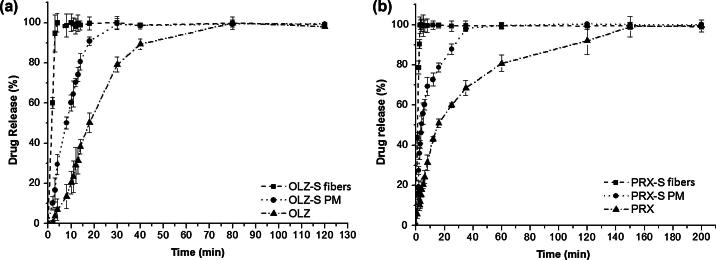
Dissolution profiles under sink conditions of (a) OLZ–sucrose fibers compared to corresponding PM and pure drug and (b) PRX–sucrose fibers compared to corresponding PM and pure drug.

Drug dissolution was initially studied under sink conditions. Fig. 7a and b compares the dissolution profiles under sink conditions of pure drugs, their corresponding PMs with sucrose and freshly prepared drug-loaded microfibers, for both OLZ and PRX. For clarity, time points in which the capsules were still intact (no drug absorbance was detected using UV) were omitted from the dissolution profiles of all samples. Significant and similar enhancements in the dissolution rates were observed for both OLZ and PRX-loaded sucrose microfibers compared to their corresponding PMs and the pure drugs. Although the rate of drug release from both PMs is increased compared to the drugs alone, the dissolution rates of drug-loaded microfibers for both drugs are very distinct from those of corresponding PMs. In particular, in the case of OLZ, the times at which 50% and 100% of drug were dissolved (*T*_50_ and *T*_100_ respectively) are observed at (1, 4), (8, 30) and (18, 80) min for OLZ-loaded microfibers, PM and pure drug respectively. Similarly for PRX, (*T*_50_, *T*_100_) values were observed at (1, 3), (4, 35) and (16, 150) min for PRX-loaded microfibers, PM and pure drug, respectively. Therefore, it is clear that the dissolution rate of drugs from microfibers is significantly enhanced compared to the PM systems. Based on these findings, there is no apparent difference between the dissolution behaviors of the two drug-loaded microfibers under sink conditions, suggesting that they should also show similar dissolution performance in vivo. Moreover, given the very rapid dissolution characteristics, high surface area and low density observed from both formulations, this approach may be potentially applicable for the development of orally disintegrating dosage forms.

#### 3.5.3 Dissolution study under non-sink conditions

**Fig. 8:**
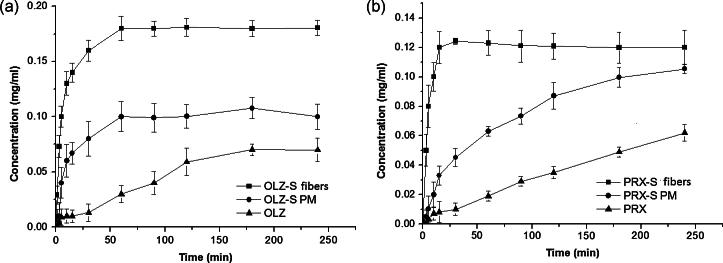
Dissolution profiles under non-sink conditions of (a) OLZ–sucrose microfibers compared to corresponding PM and pure drug and (b) PRX–sucrose microfibers compared to corresponding PM and pure drug.

Fig. 8a and b shows dissolution–supersaturation profiles obtained under non-sink conditions of the freshly prepared microfiber formulations for OLZ and PRX in comparison with corresponding pure drugs and PMs. Generally under non-sink conditions, formulations containing metastable amorphous drugs tend to generate transient supersaturated drug concentration which inevitably leads to the onset of drug recrystallization and precipitation, hence a drop in solubility. Depending on the ability of some functional excipients to act as recrystallization inhibitors, delay of drug precipitation and stabilization of relatively high apparent drug solubility can be achieved in solution [57]. This is generally related to the “spring and parachute” approach introduced by Guzmán et al., whereby the rapid initial build-up of drug supersaturation (spring profile) is maintained for a relatively long time (parachute profile) [58]. In this study, an apparent higher drug solubility compared to the corresponding pure drugs and PMs was achieved and maintained with the drug-loaded microfibers for the duration of the dissolution test (4 h) for both drugs. The absence of a drug concentration decline (i.e. maintenance of a “parachute” profile) for both drug-loaded microfibers suggests that sucrose may prevent the drugs from recrystallizing. A similar supersaturation profile was reported for tadalafil solid dispersion in HPMC prepared using freeze drying whereby drug supersaturation remained unchanged for the duration of the dissolution test due to the inhibitory effect of the carrier [7].

For OLZ, maximal dissolution was observed at 60 min for both the microfibers and the PM, although the total amount dissolved was different in the two cases: microfibers reached 0.18 mg/mL of drug concentration (equal to 90% of the total amount of drug), whereas only 0.1 mg/mL of drug concentration was reached from PMs (50% drug dissolution). The drug concentrations obtained in solution equate to roughly 3-fold and 1.4-fold supersaturation for the microfibers and PM, respectively, taking into account the solubility of OLZ in the absence of sucrose as detailed in Section 3.5.1. In both cases, the amount of drug measured in solution remained constant over the remainder of the experimental time period (4 h in total) with no indication of drug precipitation, showing the crystallization inhibition behavior of the sucrose. The difference in dissolution performance of the microfibers and the PM can be attributed to the amorphous nature and enhanced surface area of the product, as shown in the MTDSC and XRD studies.

The profile for the PRX microfiber formulation is similar to that seen with OLZ, but maximal dissolution (only 60% of the total amount of drug) was observed much earlier, at 15 min, with a −1.7-fold supersaturation. Visually, there appeared to be a slight decrease in the measured drug concentration over time, but this was not statistically significant, again showing the protective effect of sucrose against crystallization. In contrast to the case with OLZ, drug dissolution from the PRX PM was more gradual, attaining the same level as the microfibers after 4 h. However, only 16% of the total amount of drug was dissolved from the PM at 15 min, showing the superior performance of the microfibers. Again, this can be attributed to the amorphous nature and size reduction of the PRX in the microfiber formulation.

Overall, the behavior of the formulations is similar in both sink and non-sink conditions, i.e. there is a rapid initial dissolution of the drug from the microfibers with no apparent precipitation over time, and that the microfiber drug dissolution profile is significantly better, in terms of both rate and extent, than that of the PMs or the pure drugs.

It is necessary to examine the mechanisms behind the beneficial drug dissolution profile of the microfiber formulations. It has been reported that the rate of supersaturation generation, i.e. the time taken to reach maximum dissolution and supersaturation, can significantly affect the achievement of drug supersaturation and the overall time in which it is maintained [59], [60]. In particular, a high rate of supersaturation build-up generally leads to rapid nucleation and crystallization in solution. Therefore, the faster rate of supersaturation generation of PRX–sucrose microfibers compared to OLZ–sucrose microfibers would be thought to induce rapid crystallization of PRX in solution, leading to drug precipitation and loss of the initial beneficial high dissolution levels. However, this was not observed here, with the drug concentrations being maintained over the time course of both dissolution experiments for both drugs.

In addition, as previously shown in Sections 3.3, 3.4, low levels of microcrystalline PRX were found to be present in the PRX-microfiber formulation. The presence of drug crystals can act as nuclei for recrystallization, but in the present case, this does not seem to have resulted in any significant precipitation of PRX. Another aspect to consider is the potential physico-chemical interactions between carrier and drug molecules which can potentially impact on the recrystallization process during dissolution. In particular, relatively strong drug–carrier molecular interactions could potentially lead to slower drug release in the dissolution medium and therefore, slower supersaturation build-up and higher efficacy in maintaining drug supersaturation state. As an example, Chen et al. [61] carried out a comprehensive investigation of the drug–polymer–water interactions on the dissolution performance of various solid dispersion systems. Their study showed that ketoconazole/HPMC-AS solid dispersion gave the best performance due to strong drug–polymer interaction in the solid state and the strength of this interaction against water disruption.

Based on the analysis of the molecular structure of drug-loaded microfibers using ATR-FTIR reported in Section 3.4, the absence of OLZ characteristic peaks and chemical shift of ν(C–N) to a lower wavelength in the microfiber formulation suggested that hydrogen bonding between sucrose and OLZ may have occurred. In contrast, there is no evidence of formation of hydrogen bonding between PRX and sucrose in the microfiber formulation. This observation could explain the slower initial dissolution profile and supersaturation generation for OLZ–sucrose microfibers compared to PRX–sucrose microfibers. However, although there is a difference in the dissolution performance between the two drug-loaded microfiber formulations, it is noteworthy that the maximum achievable drug concentration was rapidly achieved and maintained without any drop in solubility for all the durations of the dissolution test for both drugs. This is generally identified as the main driving force to achieve successful oral absorption in which the rate of dissolution is the limiting step for systemic absorption [43], and suggests that both formulations should lead to improved oral bioavailability compared to conventional solid formulations.

## 4 Conclusions

In this study, we explore the centrifugal spinning process as a means of rapid production of solid dispersions in the form of drug-loaded microfibers with enhanced dissolution performance. Results showed that 10% OLZ- and PRX-loaded microfibers with high production yield and loading efficiency were successfully prepared using our modified centrifugal spinning machine and the quality of microfibers formed was assessed using several solid-state characterization techniques. In particular, SEM images showed microfibers with homogeneous morphology and lack of any phase separation suggesting that both drugs were successfully incorporated into the carrier.

In both sink and non-sink dissolution conditions, there was noticeable influence of the sucrose carrier on both drug solubility and dissolution rate when PMs were used compared to the pure drugs, which indicates good carrier solubilizing capacity. However, a greater drug dissolution rate and extent were obtained from the corresponding microfiber formulations which were attributed to effective reduction in drug particle size, drug amorphization and enhanced solubility of OLZ and PRX in the presence of sucrose. Both drug-loaded formulations showed rapid attainment of maximum drug dissolution with no evidence of drug precipitation over the time course of the experiment (4 h) representing the average intestinal transit time, demonstrating an effective parachute profile.

This suggests that both formulations have potential to increase the rate and extent of drug released in the absorption site, hence, improving the oral bioavailability. Therefore, overall, sugar-based drug-loaded microfibers prepared using centrifugal spinning showed great potential as an innovative formulation design in the field of solid dispersions. Moreover, on the basis of the favorable dissolution characteristics, this technique could offer a simple, potentially scalable and flexible manufacturing process to produce orally disintegrating dosage forms.

Our ongoing work is looking at critical factors involved in the incorporation of centrifugal-spun fibers into oral dosage forms. These factors include process and material parameters with a particular attention on chemical stability of formulations, mechanical properties and effect of excipient on disintegration/dissolution performance. Our approach aims to analyze these factors using a multi-scale approach whereby data at different scales (from fibers to powder to dosage forms) are collected. This approach will hopefully enable us to optimize critical factors and produce stable oral dosage forms with desirable dissolution performance while lowering the cost of product development and manufacturing.
