# Supplementary material for: Development of micro-fibrous solid dispersions of poorly water-soluble drugs in sucrose using temperature-controlled centrifugal spinning
Source: Eur J Pharm Biopharm. 2016 Jun;103:84–94. doi: 10.1016/j.ejpb.2016.03.021 (PMC4866555; doi:10.1016/j.ejpb.2016.03.021)
Supplement: Supplementary Figs. S1–S4 [file mmc1.docx]

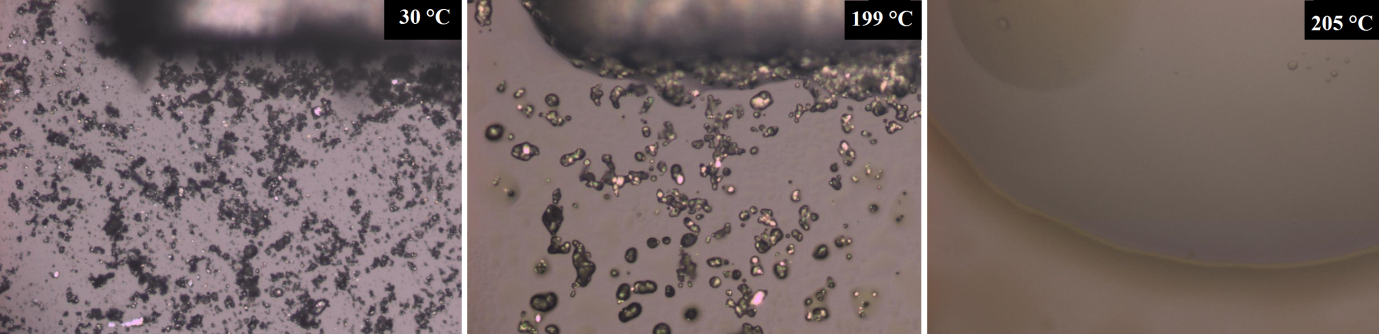


**Fig. S1.** HSM images at 10× magnification of PRX-sucrose PM captured during heating at 10 °C/min from 30 °C to the temperature when complete melting was observed.


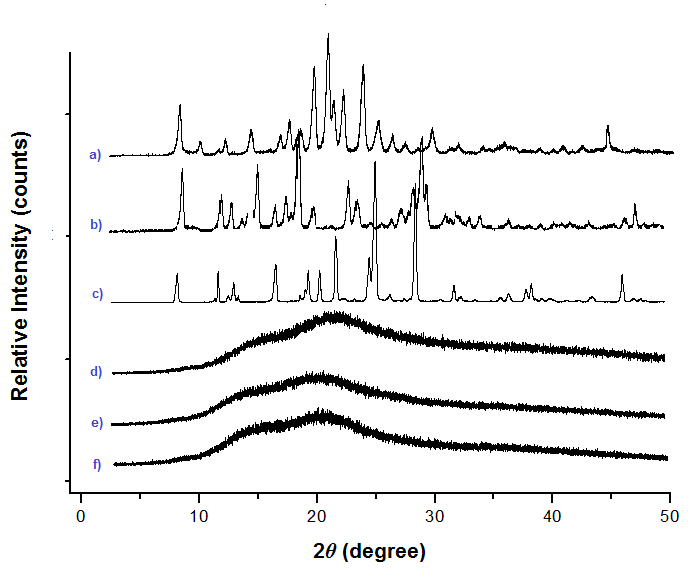


**Fig. S2.** Powder X-ray diffractograms of (a-c): sucrose, OLZ and PRX raw materials, respectively; (d-f): unloaded sucrose, 10% (w/w) OLZ-loaded sucrose and 10% (w/w) PRX-loaded sucrose microfibers respectively.


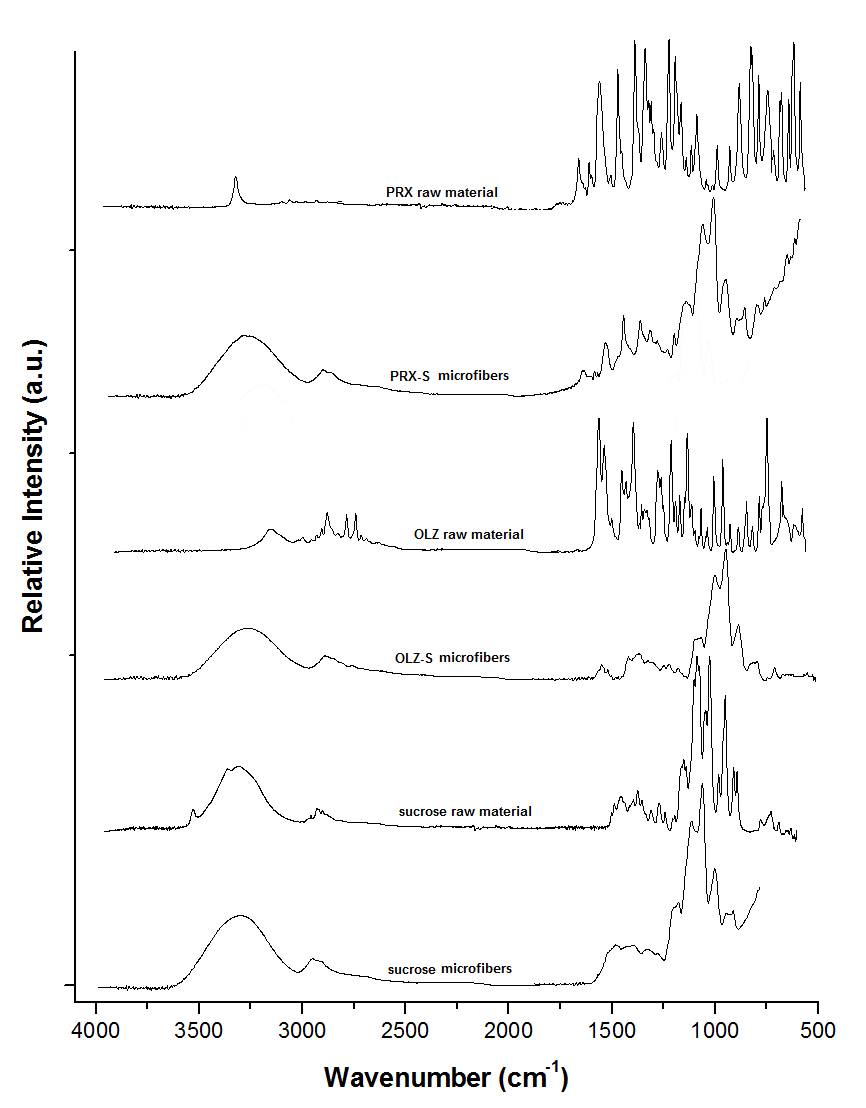


**Fig. S3**. ATR-FTIR spectra of raw materials and microfiber formulations


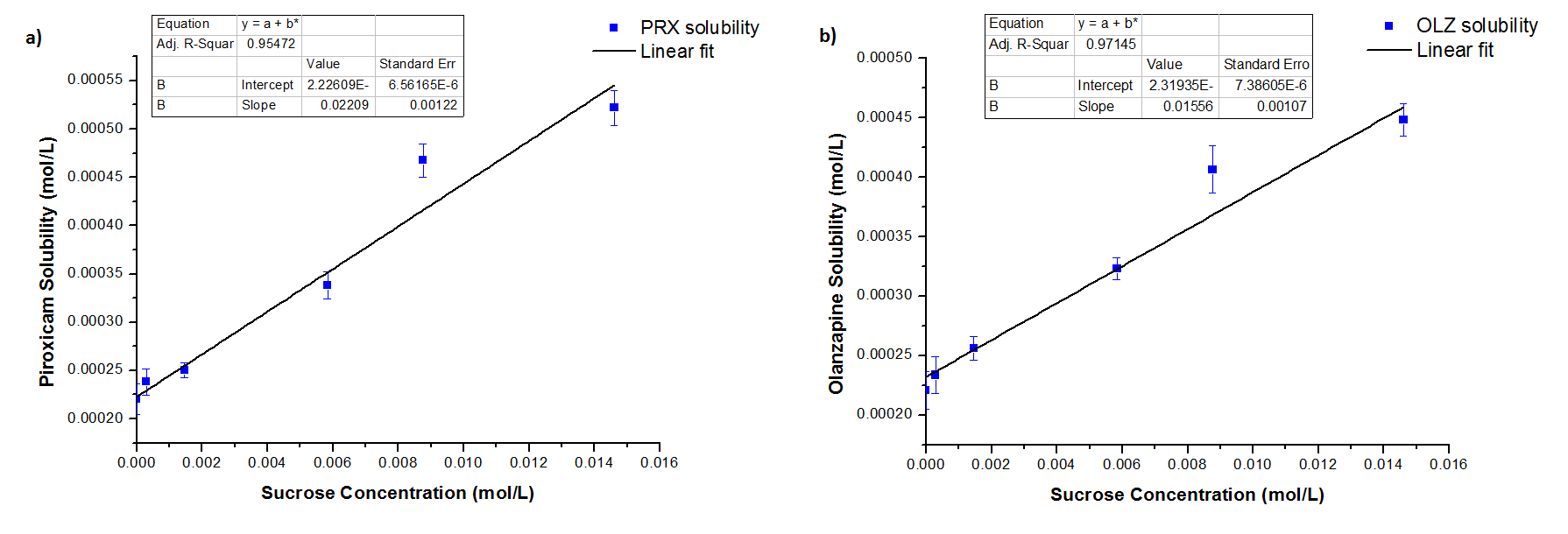


**Fig. S4**. Phase solubility diagram for (a) piroxicam and (b) olanzapine in the presence of increasing concentrations of sucrose at 37 °C
